# 

**Published:** 1894-03-03

**Authors:** 


PRICE TWOPENCE.

No. 388, Vol. XV.—March 3rd, 1894.

[Registered at the General Post Office as a Newspaper for transmission in the United Kingdom and Abroad.]

WITH EXTRA NURSING SUPPLEMENT.

**Per Annum, 8s. 6d., post free.**

**Monthly Parts, 6d.; post free, 8d.**

**Ditto per Annum, 8s., post free.**

### THE HOSPITAL

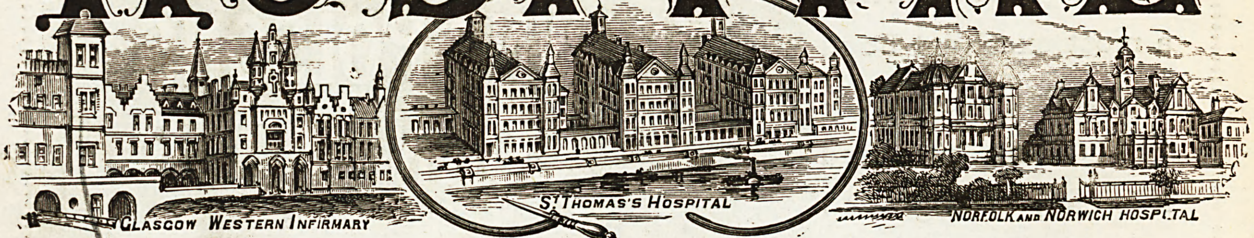

No. 388. Vol. XV. **WITH EXTRA NURSING SUPPLEMENT.** SATURDAY, MARCH 3RD, 1894.

